# Detection of Mosaic Variants in Mothers of MPS II Patients by Next Generation Sequencing

**DOI:** 10.3389/fmolb.2021.789350

**Published:** 2021-11-05

**Authors:** Alice Brinckmann Oliveira Netto, Ana Carolina Brusius-Facchin, Sandra Leistner-Segal, Francyne Kubaski, Juliana Josahkian, Roberto Giugliani

**Affiliations:** ^1^ Laboratory of Molecular Genetics, Medical Genetics Service, HCPA, Porto Alegre, Brazil; ^2^ Postgraduate Program in Genetics and Molecular Biology, UFRGS, Porto Alegre, Brazil; ^3^ National Institute on Population Medical Genetics, INAGEMP, Porto Alegre, Brazil; ^4^ BioDiscovery Laboratory, Experimental Research Center, HCPA, Porto Alegre, Brazil; ^5^ Department of Clinical Medicine, Hospital Universitario de Santa Maria (HUSM), Santa Maria, Brazil; ^6^ Department of Genetics, UFRGS, Porto Alegre, Brazil

**Keywords:** mosaicism, mucopolysaccharidosis type II, hunter syndrome, next-generation sequencing, IDS gene, x-linked disease, carrier detection

## Abstract

Mucopolysaccharidosis type II is an X-linked lysosomal storage disorder caused by mutations in the *IDS* gene that encodes the iduronate-2-sulfatase enzyme. The *IDS* gene is located on the long arm of the X-chromosome, comprising 9 exons, spanning approximately 24 kb. The analysis of carriers, in addition to detecting mutations in patients, is essential for genetic counseling, since the risk of recurrence for male children is 50%. Mosaicism is a well-known phenomenon described in many genetic disorders caused by a variety of mechanisms that occur when a mutation arises in the early development of an embryo. Sanger sequencing is limited in detecting somatic mosaicism and sequence change levels of less than 20% may be missed. The Next Generation Sequencing (NGS) has been increasingly used in diagnosis. It is a sensitive and fast method for the detection of somatic mosaicism. Compared to Sanger sequencing, which represents a cumulative signal, NGS technology analyzes the sequence of each DNA read in a sample. NGS might therefore facilitate the detection of mosaicism in mothers of MPS II patients. The aim of this study was to reanalyze, by NGS, all MPS II mothers that showed to be non-carriers by Sanger analysis. Twelve non-carriers were selected for the reanalysis on the Ion PGM and Ion Torrent S5 platform, using a custom panel that includes the *IDS* gene. Results were visualized in the Integrative Genomics Viewer (IGV). We were able to detected the presence of the variant previously found in the index case in three of the mothers, with frequencies ranging between 13 and 49% of the reads. These results suggest the possibility of mosaicism in the mothers. The use of a more sensitive technology for detecting low-level mosaic mutations is essential for accurate recurrence-risk estimates. In our study, the NGS analysis showed to be an effective methodology to detect the mosaic event.

## 1 Introduction

Mucopolysaccharidosis type II (MPS II), or Hunter syndrome (OMIM #309900) is an X-linked lysosomal storage disorder (LSD) caused by variants in the IDS gene, that encodes the iduronate-2-sulfatase enzyme. The deficiency of this enzyme lead to the accumulation of mainly two glycosaminoglycans (GAGs), dermatan sulfate and heparan sulfate, in the lysosomes, which are excreted in increased amounts in the urine (Neufeld and Muenzer, 2001). The accumulation of GAGs in multiple cells, tissues and organs, culminates in MPS II being a multisystemic disease. The most frequent clinical manifestations are skeletal abnormalities, heart disease, respiratory problems, visceromegaly, joint restriction, and, in severe cases, cognitive decline (Wraith et al., 2008). There is a broad spectrum for the phenotype that is classically divided into attenuated and severe forms, being this last one marked by progressive neurological impairment (Neufeld and Muenzer, 2001; Schwartz et al., 2007; Burton and Giugliani, 2012).

The IDS gene is located on the long arm of the X-chromosome (Xq28), comprising 9 exons and 8 introns, spanning approximately 24 kb. It was discovered by Bondenson et al. (1995) that the IDS gene has a pseudogene (IDS-2) situated approximately 20 kb from the telomeric side of the gene. The homology between the gene and the pseudogene corresponds to exons 2 and 3 and to introns 2, 3 and 7 of the IDS gene. The presence of IDS-2 makes it more susceptible for the occurrence of homologous recombination in the IDS gene (Bondenson et al., 1995b). According to the Human Gene Mutation Database v.2021.2, Public (HGMD) (Stenson et al., 2003), 626 different variants in the *IDS* gene have already been described, most of which being point mutations (49%) or small deletions (18%). As the disease has an X-linked recessive inheritance, most severely affected males do not generate offspring and homozygous females are predicted to be extremely rare (Neufeld and Muenzer, 2001).

The analysis of female carriers, in addition to detecting mutations in patients, is essential for genetic counseling, since the risk of recurrence, if the mother is heterozygous, is 50% for male children (Froissart et al., 1997). Presuming the absence of selection between carriers and non-carriers and considering that MPS II is X-linked, it is expected that approximately 1∕3 of the patients’ mothers are non-carriers and these cases are secondary to *de novo* variants (Haldane, 1935). In the work done by Chase et al. (1986), 23% of mothers of patients were identified as non-carriers, a value not that different of the expected (approximately 33%). More recent estimates are presented by Kondrashov (2003), that shows a rate of the loss-of-function mutation per locus per generation in *IDS* as 5 x 10^−6^, and by Acuna-Hidalgo et al. (2016), which brings revised data on *de novo* mutations for various diseases using next-generation sequencing techniques.

When considering diagnosis and genetic counseling, somatic mosaicism demands great commitment and can cause serious consequences if not properly detected. Commonly, the sample used for DNA analysis comes from blood, and if the mosaicism extends to this tissue, erroneous diagnosis can be provided for the patient and for the family, when it is a case for counseling (Notini et al., 2008). The diagnosis of the mothers of patients to determine if they are carriers by biochemical assays is very limited (Schröder et al., 1993). So, for the detection of carriers, several molecular biology techniques are used and the ability of Sanger sequencing to detect somatic mosaicism is limited. So, sequence change levels of less than 20% may be missed (Gajecka, 2016). Next Generation Sequencing (NGS) has been increasingly used in diagnosis. It is a sensitive and fast method for the detection of somatic mosaicism (Gajecka, 2016; Lohmann and Klein, 2014; Thorpe et al., 2020). Compared to Sanger sequencing, which represents a cumulative signal, NGS technology analyzes the sequence of each DNA read in a sample (Metzker, 2010). The Targeted Next-Generation Sequencing (TNGS) approach enables the search in different associated genes, providing greater depth of coverage and increased sensitivity and specificity (Rehm et al., 2013). This approach has been used in the past years for the diagnosis of LSDs (Fernández-Marmiesse et al., 2014), and it has also been used by our laboratory, enabling molecular genetics characterization to countless patients (Brusius-Facchin et al., 2019; Josahkian et al., 2021). This study aimed to reanalyze, by Targeted Next Generation Sequencing, 12 mothers of patients with Mucopolysaccharidosis type II that showed to be non-carriers when investigated with Sanger sequencing analysis.

## 2 Materials and Methods

### 2.1 Participants

12 mothers diagnosed as non-carriers after Sanger sequencing were selected for the reanalysis through TNGS. Genomic DNA was isolated from peripheral blood leukocytes and saliva and stored in the biorepository of the Molecular Genetics Laboratory of the Medical Genetics Service of Hospital de Clínicas de Porto Alegre (HCPA). All the samples are part of the project 13–0224, approved by the HCPA’s Institutional Review Board (IRB0000921), which is recognized by the Office for Human Research. All participants signed the MPS Brazil Network informed consent form.

### 2.2 Sanger Sequencing

Mutational analyses were carried out for the specific region of the mutation present in the index case. Polymerase Chain Reaction (PCR)-amplified products were purified and subjected to direct sequencing using ABI 3500xl 96 capillary DNA analyzer (Applied Biosystems^TM^) and the sequence was analyzed on BioEdit Sequence Alignment Editor. American College of Medical Genetics guidelines were followed for mutation nomenclature. For variant descriptions, reference sequences were NM_000,202.6 and NM_000,202.7.

### 2.3 Targeted-Next Generation Sequencing

The 12 mothers diagnosed as non-carriers after Sanger sequencing were reanalyzed through TNGS (second-tier test), using a customized panel that includes the *IDS* gene. The panel comprehends 26.75 kb, with 8 targets and 138 amplicons (Brusius-Facchin et al., 2019).

Eight of the samples were analyzed on Ion Torrent Personal Genome (PGM^TM^) System (Thermo Fisher Scientific) and the other four were analyzed on Ion GenStudio S5^TM^ System (Thermo Fisher Scientific). The samples were prepared likewise, using Ion AmpliSeq™ Library kit (Thermo Fisher Scientific), following the manufacturer’s recommendations (MAN0006943), and the quantification of the libraries was performed using Qubit® dsDNA HS kit (Thermo Fisher Scientific). The samples analyzed on the Ion PGM^TM^ had the template preparation on the Ion OneTouch2 instrument (Thermo Fisher Scientific) using the Ion PGM Template OT2 200 kit (Thermo Fisher Scientific). Attune® Acoustic Focusing Flow Cytometer (Thermo Fisher Scientific) was used to define the percentage of positive Ion Sphere Particles (ISPs), following the protocol recommendations (Part. no. 4477181). The Ion OneTouch ES (Enrichment System) was used to enrich the positive ISPs and, subsequently, the samples were loaded onto Ion 314^TM^ chip v2 (Thermo Fisher Scientific) according to the user guide (MAN0007273) and sequenced on the Ion PGM^TM^ sequencer. The samples analyzed in the Ion S5^TM^ had the template preparation on the Ion Chef^TM^ instrument (Thermo Fisher Scientific), following the manufacturer’s recommendations (MAN0016855), where the Ion 510^TM^ chip (Thermo Fisher Scientific) was loaded. Afterward, the chip was transferred to Ion S5^TM^ Sequencer and sequenced.

### 2.4 Bioinformatic Analysis

Raw data was processed and analyzed using Torrent Suite™ Software (Thermo Fisher Scientific), which imports into Ion Reporter™ Software (Thermo Fisher Scientific) a list of detected sequence variants, including SNPs and small insertions/deletions for analysis. The alignment of the sample sequence with the human genome reference 19 (Genome Reference Consortium GRCh37) was visualized and verified in the Integrative Genomics Viewer v2.3 (IGV) (Robinson et al., 2011; Thorvaldsdóttir et al., 2013). The position of the mutation present in the index case was analyzed to verify the allele frequency within this position, to conclude whether the mother had the mutation in mosaicism. Running metrics and coverage analyses were performed for the identification of technical deficiencies.

## 3 Results

### 3.1 Sanger Sequencing

**FIGURE 1:**
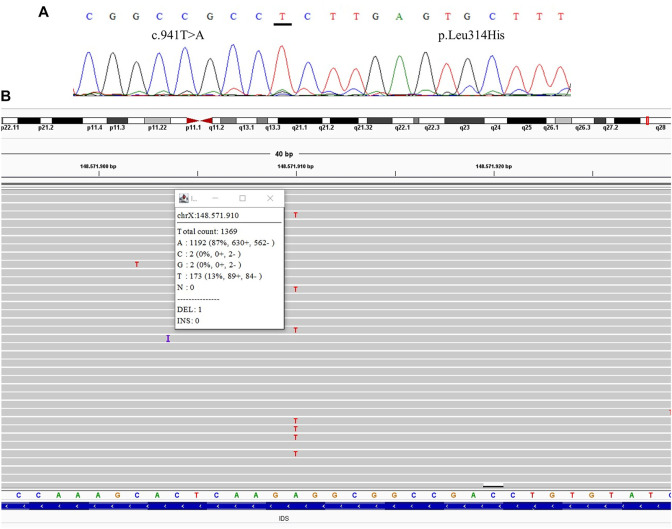
Comparative results of sanger sequencing and Next-generation sequencing at the mutation locus c.941T>A (p.Leu314His) **(A)** Sanger sequencing **(B)** Next-generation sequencing showing the coverage of the locus of the mutation, presenting the mutation at a frequency of 13%.

**FIGURE 2:**
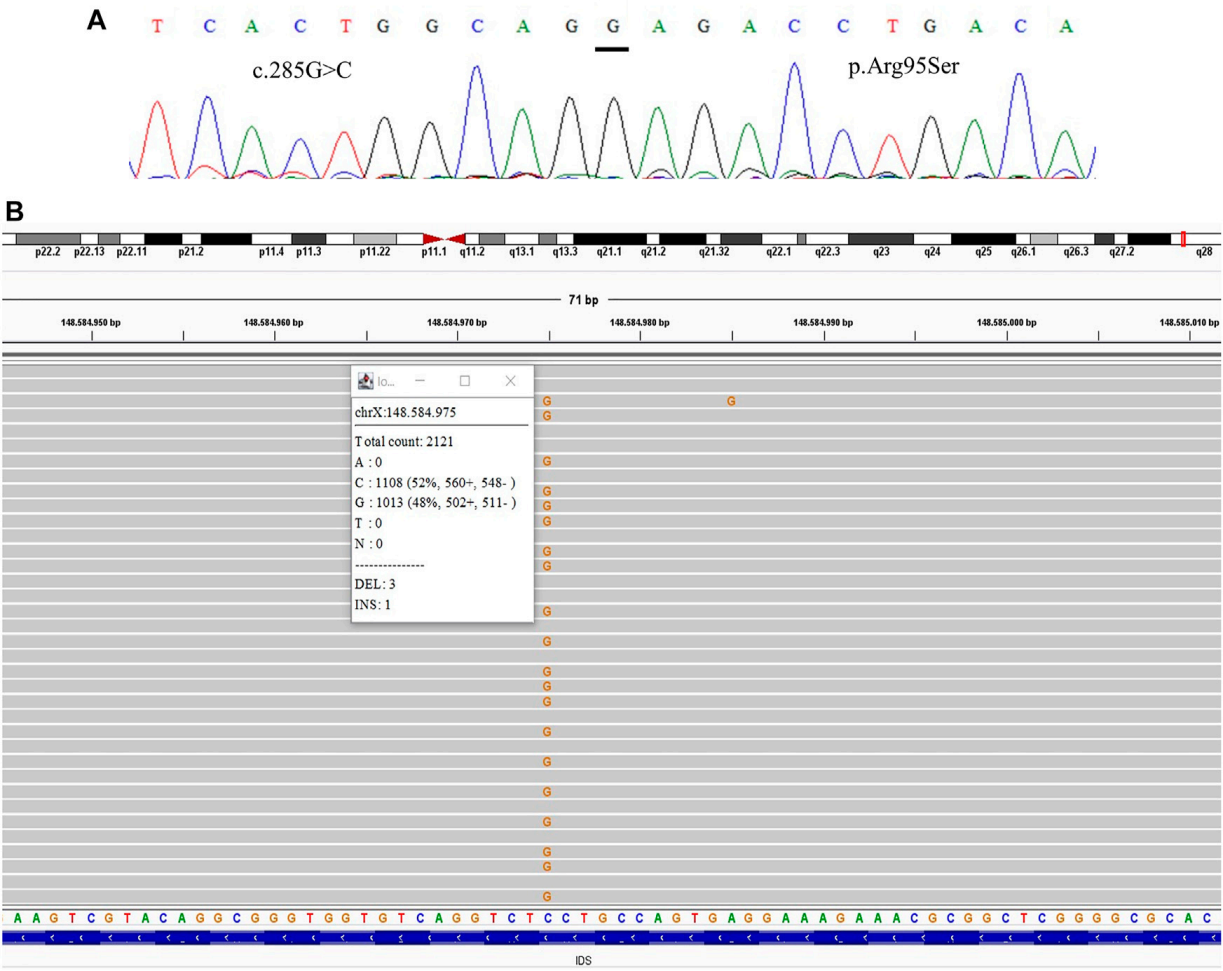
Comparative results of sanger sequencing and Next-generation sequencing at the mutation locus c.285G > C (p.Arg95Ser) **(A)** Sanger sequencing **(B)** Next-generation sequencing showing the coverage of the locus of the mutation, presenting the mutation at a frequency of 48%.

**FIGURE 3:**
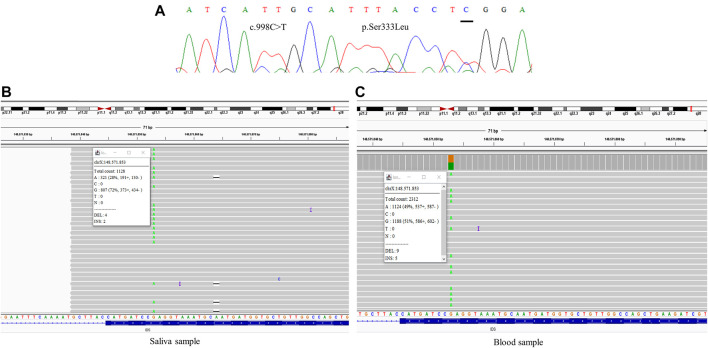
Comparative results of sanger sequencing and Next-generation sequencing at the mutation locus c.998C>T (*p*.Ser333Leu) **(A)** Sanger sequencing **(B)** Next-generation sequencing showing the coverage of the locus of the mutation in saliva sample, at a frequency of 28% **(C)** Next-generation sequencing showing the coverage of the locus of the mutation in blood sample, at a frequency of 49%.

All samples had amplified PCR products and visualization in the chromatogram. However, no signal of allele alteration in the region of the variants present in the index case was visible or it was not possible to conclude if the variant was present. Figure 1A presents the sequencing of exon 7 showing the locus of the mutation c.941T>A p.(Leu314His) demonstrating no alteration. Figure 2A presents the sequencing of exon 3 showing the locus of the mutation c.285G > C p.(Arg95Ser), demonstrating no alteration. Figure 3A presents the sequencing of exon 7 showing the locus of the mutation c.998C>T *p*.(Ser333Leu) in a chromatogram with undefined peaks, precluding a reliable analysis.

### 3.2 Targeted Next-Generation Sequencing

We were able to detect the presence of the variant previously identified in the index case in three of the mothers after the TNGS. In one case the specific variant was present in a frequency of 13% of the reads, suggesting the possibility of mosaicism in the mother (Figure 1). In another case, we found a variant present in 48% of the reads, which was still not seen in the direct sequencing (Figure 2). As the variant is in exon 3 of the IDS gene, the frequency value might be related to readings of the IDS-2 pseudogene.

One of the mothers was analyzed with samples from two different sources, saliva and blood, showing mosaicism at different levels, with frequencies ranging between 28 and 49% of the reads (Figure 3).

The mutations found in the index cases were in a variety of exons (exon 3, 6, 7, 8, and 9). They were all classified as pathogenic and with different molecular consequences, including missense, nonsense, frameshift, and alternative splicing. The mutations we were able to find in mosaicism in the mothers were in exon 3 and 7, and all of them were missense.

Running metrics and coverage analysis have shown good sequencing data, achieving 151,424 of mapped reads and 170,39 mean coverage (value obtained using the coverage 100 x of the amplicons of the IDS gene). The average of reads on target, depth of coverage, and uniformity can be seen in Table 1.

**TABLE 1:** Coverage metrics of the customized gene panel, that includes the IDS gene.

Subject	Mapped reads	Reads on target	Depth of coverage	Uniformity of coverage
1	225,826	91.26%	1,462	95.23%
2	250,405	88.90%	1,571	95.47%
3	217,933	88.67%	1,361	95.79%
4	66,682	79.26%	618.9	94.05%
5	59,097	80.49%	561.3	92.74%
6	44,284	79.70%	418.2	90.49%
7	149,116	54.90%	996.3	94.81%
8	150,089	63.25%	1,148	95.53%
9	144,869	91.11%	1,622	95.74%
10	156,075	90.58%	1,650	93.91%
11	152,194	91.29%	1,700	95.05%
12	200.52	92.68%	1,296	95.46%
Mean	151,424	82.60%	1,215	94.51%

There were other samples sequenced for the same gene panel beyond the mother’s samples that served as control, demonstrating that the detection of mosaicism was not an error.

## 4 Discussion

To the best of our knowledge, this is the first study to report mosaicism in Mucopolysaccharidosis type II through Targeted Next-Generation Sequencing. The use of a more sensitive technology for detecting low-level mosaic mutations, which may not be detectable by Sanger sequencing, is essential for accurate recurrence-risk estimates. In our study, the TNGS analysis showed to be an effective methodology to detect the event of mosaicism of Single-Nucleotide Variants (SNV), considering that we were able to identify three cases of mosaicism at different frequencies (between 13 and 49%) out of 12 that showed negative or inconclusive results in Sanger sequencing.

The fact that we did not find evidence of mosaicism in a greater number may have been because we performed the TNGS only with leukocyte DNA, which does not exclude the possibility of the occurrence of mosaicism in other tissues. In a single case, it was possible to perform the analysis with DNA extracted from saliva, as the Sanger sequencing of this case had shown undefined peaks and the mosaicism at a frequency of 49% in leukocyte DNA was an unexpected result, which motivated the analysis with a sample from a different source. As blood cells undergo several self-renewal processes in hematopoiesis, they are considered an unstable source of genetic material (Campbell et al., 2016). Furthermore, the possibility of finding somatic mosaicism should be strongly considered (Notini et al., 2008; Scarpa, 2018), by the fact that if there is no presence of mosaicism in other cell types, the *de novo* mutation rate we supposedly found (75%) would be very high compared to expected (33%) (Chase et al., 1986).

Mosaicism is a well-known phenomenon described in many genetic disorders, caused by a range of mechanisms that occur when a mutation arises in the early development of an embryo, from a unique fertilization event, producing cells with different genetic compositions (Notini et al., 2008). The mosaicism can be classified into three categories depending on the stage of development in which the mutation occurs: germline mosaicism (gonadal mosaicism), somatic mosaicism, and gonosomal mosaicism (combination of the two priors). However, it is known that a random inactivation of one of the chromosomes occurs, making only one of the chromosomes active, which is called dose compensation (Spolarics, 2007). Most of the genes present in the inactive chromosome remain inactive in all daughter cells, preserving the inactivation pattern. As a consequence of the random inactivation of the X chromosome, women are naturally mosaics, having two cell populations relative to the X chromosome, each one being expressed in one of them, being expressed on each of them, in the way that, women are mosaics for various X-linked alleles (Migeon, 2008). Besides that, all individuals are mosaics, comprising variable genotypes acquired post-zygotically (Rodríguez-Nóvoa et al., 2020). Mosaicism arises in the post-zygotic phase and since there are countless mitotic cycles to generate an adult organism (∼10^14^), many mutations arise in this step of the development (Campbell et al., 2014). These mutations can turn out to be pathogenic, but to be clinically detected they need to be present in a considered level of cells, even though they can still be transmitted to the offspring when expressed at a low cellular level (Rodríguez-Nóvoa et al., 2020). In addition, there is another phenomenon occurring in women associated with the inactivation of the X chromosome and to variants present in this chromosome. A metabolic interaction occurs between the two cell types so that women who have one copy of a mutated allele are still able to produce enough gene products with just one normal allele (Migeon, 2006). This transference of gene product between cells ends up masking the genotype of the defective cell, possibly being one of the reasons why the detection of carrier mothers by measuring the enzyme activity is unreliable. This phenomenon has been reported in X-linked lysosomal diseases such as type II Mucopolysaccharidosis and Fabry disease (Migeon, 2006).

A study by Froissart et al. (1997) showed that there are cases of mosaicism in women with a single case of MPS II in the family. The mutation was present in different frequencies for the distinct tissues analyzed, meanwhile the state of heterozygosity was found in leukocytes. Therefore, when a mutation, whether recurrent or *de novo*, is identified in a family and the mother has a molecular diagnosis of non-carrier by conventional methods, the choice of a more sensitive technique for detection of mosaic mutations (at below level), is essential for recurrence risk estimates.

As has been mentioned, mosaicism in Mucopolysaccharidosis type II has already been described by Froissart et al. (2007), in which two cases of germline associated with somatic mosaicism was described, and by Alcántara-Ortigoza (2016), in which a case of germline and somatic mosaicism was described. Likewise, there are other X-linked diseases that have reported mosaicism: X-linked Alport syndrome (Fu et al., 2016; Yokota et al., 2017; Okamoto et al., 2019; Pinto et al., 2020), X-linked retinitis pigmentosa (Strubbe et al., 2021), X-linked acrogigantism syndrome (Daly et al., 2016), Fabry disease (Barriales-Villa et al., 2019; Pianese et al., 2019), Duchenne muscular dystrophy (Winerdal et al., 2020; Dinh et al., 2018), X-linked hypophosphatemic rickets (Saito et al., 2009; Lin et al., 2020; Goji et al., 2006) and Lesch–Nyhan syndrome (Willers, 2004). Still, some of the authors managed to diagnose mosaicism in SNV also using NGS (Fu et al., 2016; Yokota et al., 2017; Barriales-Villa et al., 2019; Okamoto et al., 2019; Pinto et al., 2020; Strubbe et al., 2021)

Recent studies indicate that the occurrence of mosaicism is much more common than it was expected and that the NGS technologies, which provide high sensitivity and high throughput, help to characterize the different levels of mosaicism, including low-level mosaicism (Gajecka, 2016). Additionally, the approach of NGS using specific gene panels (TNGS), like the one used in this article, is an option that allows for higher coverage and sensitivity, with a lower cost, that has already been reported as an appropriate method to identify somatic mosaicism (Baquero-Montoya et al., 2014). It is believed that NGS is the most adequate method for the discovery of mosaicism in SNV. However, for the detection of mosaicism in Copy-Number Variants (CNVs), other methods have already been reported as more indicated, with greater sensitivity and specificity (Liu et al., 2020).

Currently, the presence of variants in mosaicism has also been considered in genetic tests performed in pre-implantation of *in vitro* embryos, called Preimplantation Genetic Testing for Monogenic Disorders (PGT-M), for couples in which there was already some evidence of the presence of pathogenic variants (Hu et al., 2021). This reinforces the importance of NGS in the detection of mosaicism, enabling a more robust molecular diagnosis and the complete information needed for genetic counseling. Likewise, there is also a study in the literature that reports the case of a mother of a patient who could be a kidney donor that was re-evaluated and found to be a carrier of the index case mutation in mosaicism (Pinto et al., 2020), bringing another case in which the NGS enabled the complete diagnosis.

To this extent, we believe that the NGS analysis, using the TNGS approach, showed to be an effective methodology to detect the mosaic event, in different levels (13–49%), in the mothers of affected patients with Mucopolysaccharidosis type II. Since failure to identify low levels of mosaic mutations may lead to the misinterpretation of molecular results, especially for a carrier, the analysis with sensitive methods such as TNGS is very important for complete diagnostic and genetic counseling.

## Data Availability

The datasets presented in this article are not readily available due to ethical and privacy restrictions. Requests to access the datasets should be directed to the corresponding author.
